# Survey Fatigue During the COVID-19 Pandemic: An Analysis of Neurosurgery Survey Response Rates

**DOI:** 10.3389/fsurg.2021.690680

**Published:** 2021-08-12

**Authors:** Rosaline de Koning, Abdullah Egiz, Jay Kotecha, Ana Catinca Ciuculete, Setthasorn Zhi Yang Ooi, Nourou Dine Adeniran Bankole, Joshua Erhabor, George Higginbotham, Mehdi Khan, David Ulrich Dalle, Dawin Sichimba, Soham Bandyopadhyay, Ulrick Sidney Kanmounye

**Affiliations:** Department of Research, Association of Future African Neurosurgeons, Yaoundé, Cameroon

**Keywords:** COVID-19, neurosurgery, survey fatigue, survey, response rate, non-response, data collection

## Abstract

**Background:** The COVID-19 pandemic has caused a surge in research activity while restricting data collection methods, leading to a rise in survey-based studies. Anecdotal evidence suggests this increase in neurosurgical survey dissemination has led to a phenomenon of survey fatigue, characterized by decreased response rates and reducing the quality of data. This paper aims to analyze the effect of COVID-19 on neurosurgery surveys and their response rates, and suggest strategies for improving survey data collection.

**Methods:** A search was conducted on March 20, 2021, on Medline and EMBASE. This included the terms “neurosurgery,” “cranial surgery,” “spine surgery,” and “survey” and identified surveys written in English, on a neurosurgical topic, distributed to neurosurgeons, trainees, and medical students. Results were screened by two authors according to these inclusion criteria, and included articles were used for data extraction, univariable, and bivariable analysis with Fisher's exact-test, Wilcoxon rank-sum test, and Spearman's correlation.

**Results:** We included 255 articles in our analysis, 32.3% of which were published during the COVID-19 pandemic. Surveys had an average of 25.6 (95% CI = 22.5–28.8) questions and were mostly multiple choice (78.8%). They were disseminated primarily by email (75.3%, 95% CI = 70.0–80.6%) and there was a significant increase in dissemination *via* social media during the pandemic (OR = 3.50, 95% CI = 1.30–12.0). COVID-19 surveys were distributed to more geographical regions than pre-pandemic surveys (2.1 vs. 1.5, *P* = 0.01) and had higher total responses (247.0 vs. 206.4, *P* = 0.01), but lower response rates (34.5 vs. 51.0%, *P* < 0.001) than pre-COVID-19 surveys.

**Conclusion:** The rise in neurosurgical survey distribution during the COVID-19 pandemic has led to survey fatigue, reduced response rates, and data collection quality. We advocate for population targeting to avoid over-researching, collaboration between research teams to minimize duplicate surveys, and communication with respondents to convey study importance, and we suggest further strategies to improve response rates in neurosurgery survey data collection.

## Introduction

During the Coronavirus Disease (COVID-19) pandemic, many global organizations have strived to conduct research to find solutions and mitigation strategies to the burdens of the pandemic (1). Indeed, the National Institute of Health estimates more than a fifth of the current biomedical community have pivoted their efforts to address COVID-19 related research questions - showing impressive adaptability of the research community (2, 3) - and overall publications have hit a record high (4). However, the pandemic has affected how research in and of itself is conducted, and the feasibility of conducting non-COVID-19 related studies. For instance, new subject enrollment for clinical trials across all specialties dropped by 79% between April 2019 and April 2020, and by 76% in neurological and neurosurgical studies (5). Social distancing and quarantine rules have delayed clinical trials and laboratory work so academics have been forced to embrace remote strategies for primary data collection (6). Therefore, online surveys have become a crucial tool during the COVID-19 pandemic, allowing for the collection of real-time data despite the global restrictions that have been put in place (7). Online surveys come with a host of advantages: ease of use for the respondent, ease of data analysis for the surveyor, low cost, wide range of options for dissemination, and flexibility of question design (8).

However, the authors' anecdotal experience in the neurosurgical field indicates that an increased propensity for survey fatigue has accompanied the recent surge in survey dissemination. Survey fatigue is a well-known phenomenon in academia, occurring when respondents tire of the survey they're completing and produce suboptimal responses or terminate participation pre-maturely (9). This leads to an overall lower quality of respondent data and reduces the power of studies conducted through this method of data collection. A number of factors are known to influence respondent fatigue, such as survey length, survey topic, question complexity, and question type (10), and literature has been published with advice on minimizing the chances of fatigue occurring (11, 12).

In this paper, we propose a second type of survey fatigue, characterized by lower response rates. This proposition is driven by the experiences from a recent unpublished collaborative between the Neurology and Neurosurgery Interest Group (NANSIG) and the Association of Future African Neurosurgeons (AFAN), where despite survey reminders, social media dissemination, and extension of data collection period, the survey was met with only 13 responses from across the continent of Africa (unpublished data). We believe that the surge in survey dissemination in the neurosurgical field since the beginning of the COVID-19 pandemic has led to potential survey respondents being approached more frequently within a short period, leading to a type of survey fatigue in which these respondents refuse to complete surveys at all. There is currently no data available in the neurosurgical literature quantifying the changes in survey dissemination and response in light of the COVID-19 pandemic, and understanding these changes and the drivers behind them is crucial to improve the quality of data collection in future studies.

This study aims to analyze the effects of the COVID-19 pandemic on neurosurgical survey production and responses. We will analyze the number, pattern of distribution, and response rates of surveys produced before the pandemic and since the pandemic to investigate changes between these two time periods, and understand how COVID-19 has impacted neurosurgical data collection through surveys. We will particularly be looking at the presence of survey fatigue. Finally, we will investigate what factors may have contributed to the development of survey fatigue within the neurosurgical community during the COVID-19 pandemic, to inform future survey design and improve the quality of data collected.

## Methods

A search strategy was developed to capture all surveys completed by neurosurgery attendings/consultants, residents/registrars, and medical students interested in the specialty. The databases MEDLINE and Embase were searched using key terms such as “neurosurgery,” “spine surgery,” “cranial surgery,” and “survey” from inception to March 20, 2021. The results were screened by two authors (RdK and USK) according to our inclusion criteria, using the software Rayyan (13). For the article to be included for data extraction, the article had to be (i) written in English, (ii) detailing a survey administered to neurosurgeons, neurosurgical trainees, or students interested in neurosurgery, (iii) and the survey had to be based on a topic relevant to neurosurgery (i.e., surveys exploring general medical or surgical concepts relevant to other specialties were excluded).

The included articles were then used for data extraction, in which they were segregated into surveys conducted before the COVID-19 pandemic, and surveys conducted since the start of the COVID-19 pandemic. Numbers of surveys, respondents, and response rates were then analyzed for each of these groups. Summary descriptive statistics were calculated for qualitative (i.e., frequencies and percentages) and quantitative (i.e., mean and 95% confidence interval) data. Then, bivariable analysis was computed using: Fisher's exact-test, odds ratios and their 95% confidence intervals; Wilcoxon rank-sum test; and Spearman's correlation.

## Results

**Figure 1:**
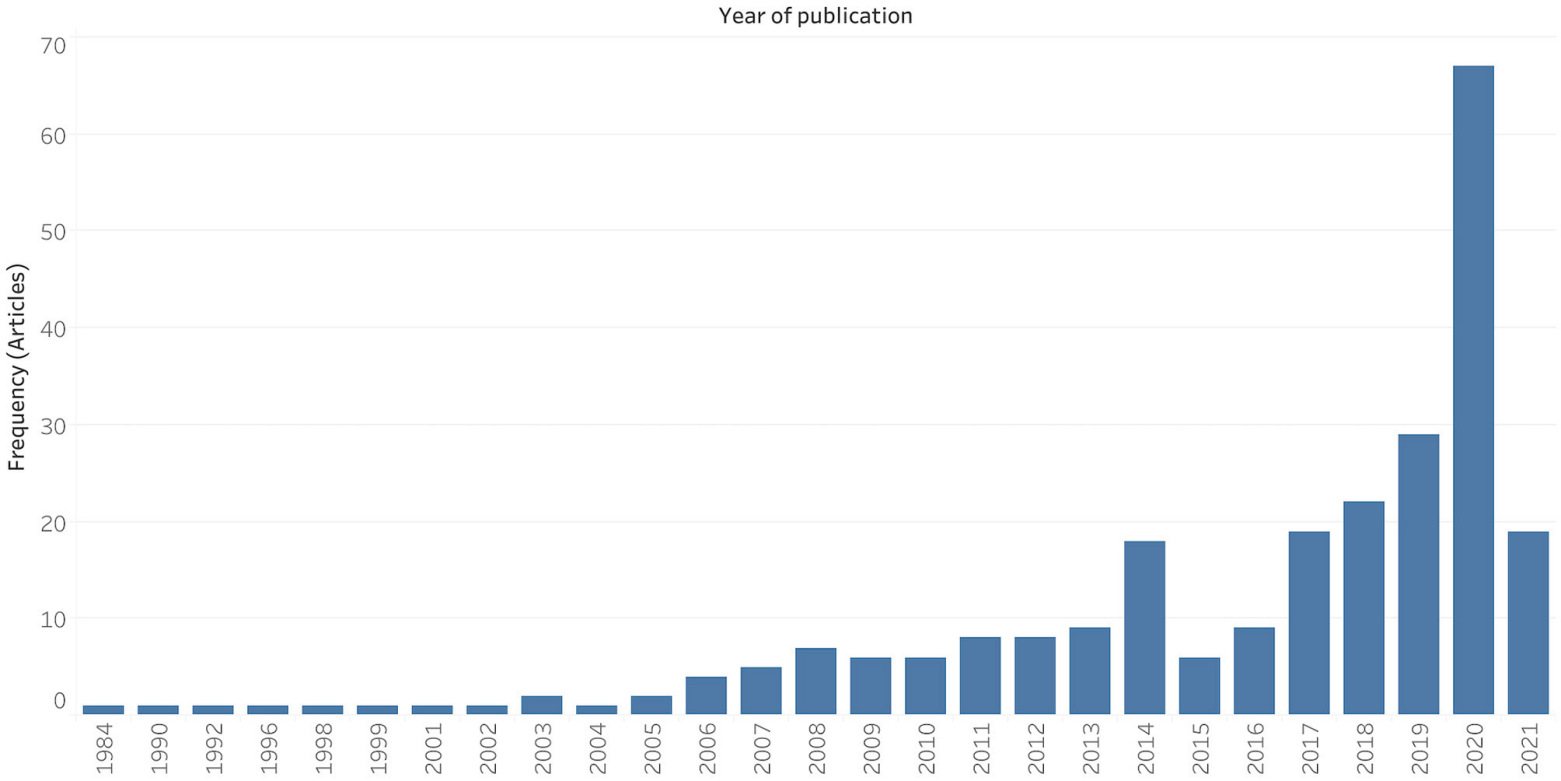
Publication trend of neurosurgery survey research.

Two hundred and fifty-five studies met the inclusion criteria. They were published between 1984 and 2021, with 67.7% (*n* = 172) of papers published in the pre-COVID-19 era. The COVID-19 era was defined as beginning on the 1st of January 2020, as per the WHO report on December 31st, 2019 (14). There was a clear publication peak in 2020 (*n* = 67, 26.3%), corresponding to the year of the COVID-19 pandemic (Figure 1).

Each survey collected responses from an average of 1.7 (95% CI = 1.49–1.90) WHO regions and 1.2 (95% CI = 1.1–1.2) professional groups (i.e., medical students, residents/registrars, or neurosurgeon consultants/attendings). Overall, COVID-19 era surveys were distributed to more geographical regions than pre-COVID-19 era surveys (2.1 vs. 1.5, *P* = 0.01). We found that more neurosurgery surveys of Southeast Asian (OR = 3.22, 95% CI = 1.64–7.00, *P* = 0.001) and Eastern Mediterranean (OR = 2.39, 95% CI = 1.13–5.42, *P* = 0.03) respondents were published in the COVID-19 era than in the pre-COVID-19 era. There was no evidence to suggest differences in the number of surveys targeting medical students, residents, and attendings between the two timeframes (Table 1).

**Table 1:** Neurosurgery survey study characteristics in the pre-COVID-19 and COVID-19 eras.

**Study population**	**Pre-COVID-19 era** **frequency (percentage)** ***n* = 172 (67.7)**	**COVID-19 era** **frequency (percentage)** ***n* = 83 (32.3)**	**Odds ratio** **(95% CI)**	***P*-value** **Fisher's exact**
**Professional role**				
Attendings	135 (52.9)	55 (21.6)	0.56 (0.31–1.00)	0.06
Residents	60 (23.5)	28 (11.0)	0.97 (0.56–1.68)	0.99
Medical students	9 (3.5)	7 (2.7)	1.69 (0.61–4.71)	0.41
**Region**				
Africa	33 (12.9)	22 (8.6)	1.53 (0.80–2.92)	0.24
America	88 (34.5)	43 (16.9)	0.98 (0.54–1.85)	0.99
Europe	39 (15.3)	26 (10.2)	1.58 (0.84–2.94)	0.15
Eastern Mediterranean	17 (6.7)	17 (6.7)	2.39 (1.13–5.42)	0.03^*^
South East Asia	18 (7.1)	22 (8.6)	3.22 (1.64–7.00)	0.001^*^
Western Pacific	19 (7.5)	16 (6.3)	1.94 (0.92–4.47)	0.11
**Question types**				
Multiple-choice	126 (49.4)	62 (24.3)	0.98 (0.28–4.10)	0.99
Free text	43 (16.9)	27 (10.6)	1.47 (0.80–2.73)	0.27
**Mode of distribution**				
Email	111 (43.5)	53 (20.8)	0.85 (0.42–1.89)	0.70
Social media	8 (3.1)	12 (4.7)	3.50 (1.30–12.0)	0.01^*^
Post mail	16 (6.3)	4 (1.6)	0.48 (0.11–1.20)	0.22
Oral	7 (2.7)	3 (1.2)	0.86 (0.22–3.65)	0.99

*Percentage of each study characteristic relative to the total 255 articles are presented*.

* *P < 0.05*.

On average, the surveys were composed of 25.6 (95% CI = 22.5–28.8) questions. The majority of studies were made up of multiple-choice questions (*n* = 201, 78.8%) and 77 surveys (30.2%) contained free-text questions. Each survey had an average of 1.3 (95% CI = 1.2–1.4) question types (i.e., multiple choice, free text, Likert scale, and short answer) and was distributed by a single mode (95% CI = 1.0–1.1). The survey distribution modes included email (*n* = 192, 75.3%, 95% CI = 70.0–80.6%), post-mail (*n* = 26, 10.2%, 95% CI = 6.5–13.9%), social media (*n* = 20, 7.8%, 95% CI = 15.1–24.9%), and oral distribution (*n* = 12, 4.7%, 95% CI = 2.1–7.3%). There was a three-fold increase in dissemination *via* social media in the COVID-19 era in comparison to the pre-COVID-19 era (OR = 3.50, 95% CI = 1.30–12.0).

The surveys collected an average of 194.9 (95% CI = 157.3–232.5) total responses, and they had a mean response rate of 44.7% (95% CI = 40.3–48.9%). The greater the number of regions surveyed, the higher the number of responses (*R* = 0.26, *P* < 0.001). The response rate negatively correlated with the number of regions surveyed (*R* = −0.24, *P* = 0.001) and with the total number of responses (*R* = −0.40, *P* < 0.001) (Table 2).

**Table 2:** Correlation between total number of responses or response rates and quantitative independent variables.

**Quantitative variables**	**Correlation** **coefficient**	***P*-value** **Spearman's rho**
**Total number of responses**		
Number of regions surveyed	0.26	<0.001^**^
Number of study populations	0.01	0.92
Response rate	−0.40	<0.001^**^
Total number of questions	0.04	0.56
Number of question types	−0.12	0.09
Number of survey distribution modes	−0.04	0.50
**Response rate**		
Number of regions surveyed	−0.24	0.001^*^
Number of study populations	−0.03	0.69
Total number of responses	−0.40	<0.001^**^
Total number of questions	0.05	0.53
Number of question types	0.10	0.18
Number of survey distribution modes	0.001	0.99

* *P < 0.01*,

** *P < 0.001*.

Studies collecting data from America (−18.5%, *P* < 0.001), the Western Pacific (−15.8%, *P* = 0.03), Southeast Asia (−15.1%, *P* = 0.03), and the Eastern Mediterranean (−13.6%, *P* = 0.01) regions had lower response rates in the COVID-19 era (Table 3). Overall, COVID-19 era surveys had higher total responses (247.0 vs. 206.4, *P* = 0.01) but lower response rates (34.5 vs. 51%, *P* < 0.001) than pre-COVID-19 era surveys.

**Table 3:** Response rates across various study populations.

**Study populations**	**Mean response** **rate difference**	***P*-value** **Wilcoxon rank-sum** **test**
**Professional role**		
Attendings	−1.5%	0.78
Residents	−0.5%	0.69
Medical students	+3.5%	0.54
**Region**		
Africa	−2.3%	0.52
America	−18.5%	<0.001^**^
Europe	+2.1%	0.88
Eastern Mediterranean	−13.6%	0.01^*^
South East Asia	−15.1%	0.003^*^
Western Pacific	−15.8%	0.003^*^

* *P < 0.01*,

** *P < 0.001*.

## Discussion

### Key Findings

In this study, we analyzed the effects of the COVID-19 pandemic on the pattern of neurosurgical survey production and responses. There was a significant increase in the number of neurosurgical surveys published since the COVID-19 pandemic. There was a particular increase in surveys of Southeast Asian and Eastern Mediterranean respondents during the COVID-19 pandemic. The primary mode of survey dissemination was email, and there has been a three-fold increase in survey dissemination through social media since the beginning of the pandemic. We found that COVID-19 era surveys were distributed to more regions, and while surveying more regions led to a greater number of responses, it was also associated with a lower response rate. Overall, surveys conducted during the COVID-19 pandemic were found to have a higher total number of responses but lower response rates.

### Implications

These results support our hypothesis that the COVID-19 pandemic has led to survey fatigue characterized by non-response (respondents refusing to complete any part of a survey), as reflected by the lowered response rate during the pandemic. During the COVID-19 pandemic, the number of surveys created and disseminated has increased significantly, and on average, each survey has targeted more regions. Therefore, more neurosurgical attendings/consultants, residents/registrars, and interested medical students have been solicited for surveys now than ever before, and all within a very brief window of time. Thus, non-response survey fatigue may be a consequence of individuals feeling overwhelmed with the number of survey requests. This is of particular note if that individual is a member of a small sample population, such as members within the neurosurgical field. For example, there are approximately only 500 neurosurgeons across Sub-Saharan Africa (15), and so any studies which attempt to survey this population are limited to a small number of potential responders. Each surgeon may therefore receive multiple requests occurring simultaneously or in close succession, leading to a feeling of being “over-researched” (16). This feeling of being over-researched may be further exacerbated if the content of surveys overlaps (16). In order to prevent such repetition and duplication of efforts, researchers should consult repositories of protocols for ongoing studies and discuss new studies with those who are likely to be aware of potential overlaps or synergies. This may include professional organizations, research funders, and government agencies (17).

Furthermore, the three-fold increase in social media dissemination during the COVID-19 pandemic can contribute to this feeling by creating the illusion that survey requests are omnipresent. To tackle this, researchers could be encouraged to receive training on the use of social media for participant recruitment. This training should highlight how to utilize social media to reach a population of interest, whilst minimizing spread to individuals for whom the survey is irrelevant. Relevant users could be identified through previous activity and interests, for example (18). It should also be noted that although social media dissemination is effective at recruiting participants for studies across a range of specialties (19), its use also limits the pool of participants to those neurosurgeons with access to the internet and social media platforms. This is particularly relevant to our unpublished survey, which was disseminated across Africa. Prior literature has shown a reduced response rate to online surveys amongst healthcare professionals from low- and middle-income countries (LMICs). Proposed reasons for this included inconsistent internet connection, more expensive mobile data, and reduced time to respond to surveys due to increased patient care workload (10).

It may also be that respondents from LMICs do not feel confident in filling out surveys on topics they are unfamiliar with, which is more likely to occur in cases where surveys are distributed globally to both high-income countries and LMICs. Reduced understanding of the questionnaire has been shown to correlate with higher levels of non-response (20). If surveys distributed globally consistently present topics that are unfamiliar to LMIC clinicians, it could lead to LMIC clinicians believing that future surveys sent out to a global audience are likely to be irrelevant, and therefore should be ignored (21). This eventuality would hamper trans-national efforts centered around health equity and information sharing. Therefore, it is critical when designing research studies that the applicability of the content for an international audience is taken into consideration. This could even be mandated at the ethical approval stage or through the creation of gatekeepers for research conducted internationally. Possible gatekeepers are organizations that represent clinicians. Approval of a survey from local, state or national organizations has also been shown to improve physician response (22). Therefore, gatekeepers can improve trust amongst physicians and prompt a higher response rate (23). Gatekeepers are also likely to reduce dissemination of similar survey projects and can prevent survey fatigue in this way.

In addition to over-researching and technological challenges, lack of communication with participants to convey the importance of their responses can also discourage engagement with surveys, as it is more difficult for participants to appreciate the relevance of their contributions and feel invested in the study (16). Lack of investment into the results of the survey has been shown to contribute to non-response (24, 25). Offering to share the survey results provides an opportunity to discuss the importance of the survey and creates a more trustful relationship between the surveyor, and participant and can stimulate the participant to take an active interest into the importance of the survey project. This also allows for a potential avenue for participant feedback once the results of the survey have been disseminated to each participant, which could help improve future projects (17).

Table 4 provides further evidence-based recommendations to improve response rates and reduce fatigue in surveyed populations. These interventions relate to both survey design and dissemination, and along with the above recommendations, increase the likelihood of potential respondent engagement with neurosurgery surveys, allowing for the collection of higher quality data.

**Table 4:** Interventions proven to increase response rates.

**Intervention**	**Explanation**
Keep the questionnaire brief	Shorter questionnaires require less time to complete, reducing respondent burden (25)
Use simple and precise language	Poor wording of questions and lack of clarity reduces respondent motivation (26)
Provide a personalized invitation	Personalization decreases the perception of anonymity and increases investment (27)
Translate the survey into relevant languages	Response rates increase when respondents can complete the survey in their mother tongue (28)
Set a deadline for responses	This creates an illusion of urgency that helps encourage responses (23)
Send regular reminders	Reminders increase survey visibility and likelihood of response (29)
Offer a financial incentive	Respondents are more likely to engage when something is promised in return (30)

### Limitations

One limitation of our study is that we were unable to extract data about all variables that may contribute to survey fatigue and non-response, either because included papers did not provide the adequate information, or because analysis was not possible. For example, the length of the data collection period was too variable across studies to analyze its relationship with total responses and response rates, so we were unable to determine whether this has an effect. Survey quality may also affect response rate, but many papers included in this study did not provide a copy of the questionnaire, preventing us from formally appraising this potential contributing factor. Another limitation is that of the nature of publication: some surveys will inevitably not have been published, and others might still be in review or writing. However, we believe that this latter point would only support our results, as the surveys as yet unpublished will more likely have been conducted recently, since the beginning of the COVID-19 pandemic.

## Conclusion

The COVID-19 pandemic has caused unprecedented challenges to primary data collection through clinical trials and laboratory research, leading to a significant rise in online strategies, such as neurosurgical survey distribution. The results from this study confirm our hypothesis that this surge in survey production has also led to the development of a phenomenon known as survey fatigue, characterized by reduced response rates. We've suggested a number of methods to tackle this problem, and thus improve the quality of data collected through surveys. Mindful population targeting prevents respondents from feeling over-researched, collaboration between research teams minimizes duplication of survey questions, and communication with respondents can convey study importance to incentivize potential respondents to participate in neurosurgical surveys.

## Data Availability Statement

The raw data supporting the conclusions of this article will be made available by the authors, without undue reservation.

## Author Contributions

RdK: conception, methodology, data extraction, writing, and editing. AE, JK, and AC: data extraction, writing, and editing. SO, NB, JE, and MK: data extraction and writing. GH and SB: writing and editing. DD: writing. DS: visualization. UK: conception, methodology, data analysis, and editing. All authors contributed to the article and approved the submitted version.

## Conflict of Interest

The authors declare that the research was conducted in the absence of any commercial or financial relationships that could be construed as a potential conflict of interest.

## Publisher's Note

All claims expressed in this article are solely those of the authors and do not necessarily represent those of their affiliated organizations, or those of the publisher, the editors and the reviewers. Any product that may be evaluated in this article, or claim that may be made by its manufacturer, is not guaranteed or endorsed by the publisher.
